# Follicular lymphoma in the modern era: survival, treatment outcomes, and identification of high-risk subgroups

**DOI:** 10.1038/s41408-020-00340-z

**Published:** 2020-07-17

**Authors:** Connie L. Batlevi, Fushen Sha, Anna Alperovich, Ai Ni, Katy Smith, Zhitao Ying, Jacob D. Soumerai, Philip C. Caron, Lorenzo Falchi, Audrey Hamilton, Paul A. Hamlin, Steven M. Horwitz, Erel Joffe, Anita Kumar, Matthew J. Matasar, Alison J. Moskowitz, Craig H. Moskowitz, Ariela Noy, Colette Owens, Lia M. Palomba, David Straus, Gottfried von Keudell, Andrew D. Zelenetz, Venkatraman E. Seshan, Anas Younes

**Affiliations:** 1 Department of Medicine, Lymphoma Service, Memorial Sloan Kettering Cancer Center, New York, NY USA; 2 Department of Epidemiology and Biostatistics, Memorial Sloan Kettering Cancer Center, New York, NY USA; 3 Present Address: Division of Biostatistics, College of Public Health, Ohio State University, Columbus, OH USA; 4 Present Address: The Royal Marsden Hospital, London, UK; 5 Present Address: Peking University Cancer Hospital, Beijing, China; 6 Present Address: Center for Lymphoma, Massachusetts General Hospital Cancer Center, Boston, MA USA; 7 Present Address: Sylvester Comprehensive Cancer Center, University of Miami Health System, Miami, FL USA

**Keywords:** Targeted therapies, Risk factors, Medical research, B-cell lymphoma, Disease-free survival

## Abstract

Patients with follicular lymphoma (FL) frequently require multiple treatments during their disease course; however, survival based on lines of treatment remains poorly described in the post-rituximab era. Also, the Follicular Lymphoma International Prognostic Index (FLIPI) score was developed to predict survival at diagnosis, yet it remains unknown whether increase in FLIPI score following an initial observation period is associated with less-favorable outcomes. To address these knowledge gaps, we retrospectively studied 1088 patients with FL grade 1–3A managed between 1998 and 2009 at our institution. Median overall survival (OS) and progression-free survival (PFS) after first-line treatment were not reached and 4.73 years, respectively. Following successive lines of treatment, years of median OS and PFS were, respectively: after second-line, 11.7 and 1.5; third-line, 8.8 and 1.1; fourth-line, 5.3 and 0.9; fifth-line, 3.1 and 0.6; sixth-line, 1.9 and 0.5. In initially observed, subsequently treated patients, FLIPI score increase after observation was associated with inferior survival following first-line treatment. The reduced survival we observed after second-line and later therapy supports the development of new treatments for relapsed patients and benchmarks historical targets for clinical endpoints. This study also highlights the utility of changes in FLIPI score at diagnosis and after observation in identifying patients likely to have worse outcomes.

## Introduction

Follicular lymphoma (FL) is the second most common lymphoma in the United States, with approximately 14,000 patients being diagnosed each year^1,2^. While FL remains incurable, overall survival (OS) continues to improve due to improvements in diagnostic tools and supportive care, the development of the monoclonal anti-CD20 antibody rituximab, and the increasing number of FDA-approved therapies^3–5^. Current first-line regimens for FL typically achieve high response rates^6–12^. As the disease recurs, patients are treated with multiple lines of therapies during their lifetime. The outcome of these different regimens and the impact on patient survival remain understudied in the modern era. A secondary analysis from the LymphoCare study recently reported progression-free survival (PFS) but not OS for first- through fifth-line therapy^13^. We investigated how the survival outcomes OS, PFS, and event-free survival (EFS) evolved after multiple lines of therapy, information that should aid in estimating clinical endpoints when designing clinical trials for multiply-relapsed patients. Another aim of this study was to provide treating physicians with additional biomarkers predictive of high-risk patients that might permit identification for early treatment intervention.

Prior studies have shown patients with FL with low tumor burden can be initially observed without impacting survival^14,15^. Even so, patients who undergo initial observation follow a heterogenous clinical course. The FL International Prognostic Index (FLIPI), a five-factor risk model based on age, stage, lactate dehydrogenase and hemoglobin levels, and number of nodal areas, has been validated as a diagnostic model in both the pre- and post-rituximab eras. It is used to predict patient survival and to stratify patients in clinical trials^4,16^. However, many patients are initially observed and have prolonged lead time from diagnosis to first treatment. Moreover, there are no data on the stability of FLIPI score in initially observed patients, nor the impact of changes in FLIPI score during initial observation on survival or the rate of subsequent histological transformation to other types of lymphoma. Here we investigate, for patients who were initially observed, whether FLIPI risk group had changed between time of diagnosis and the time of initial treatment, and whether increased FLIPI score impacted outcomes after treatment.

## Subjects and methods

### Study design and patients

We retrospectively examined outcomes for patients diagnosed during the years 1998−2009 with de novo FL managed at Memorial Sloan Kettering Cancer Center (MSK). The beginning year was selected to capture patients with exposure to rituximab, FDA-approved for non-Hodgkin’s lymphoma in 1997; end year was chosen to ensure at least 10-year follow-up. The institutional review board approved this retrospective analysis; all patients had given written informed consent to biospecimen protocols. We excluded patients who were <18 years old; were diagnosed with an active concurrent malignancy; had grade 3B FL at diagnosis because of it’s management similar to diffuse large B cell lymphoma; or whose pathology showed composite histology at diagnosis. We also excluded patients who were managed with fewer than three visits at our institution, indicative of a consultative role; but not patients who had died before their third visit. At least one pathology specimen for each patient with FL was centrally reviewed at our institution. Documentation of transformations to other forms of lymphoma was based on biopsy confirmation. Clinical staging and best overall response, as assessed by treating physicians, were extracted from chart review. Ambiguity in staging or response to therapy was settled by review of available radiographic and pathologic reports.

Intention of treating physician to manage certain patients with active surveillance was assessed by medical record review. Patients whose first-line treatment began ≥12 months after diagnosis were considered initially observed. For all other patients, charts were reviewed to determine the physician’s intent of observation or initial treatment.

All authors had access to the study data. Chart review was performed by C.L.B., A.A., F.S., K.S., J.S., and Z.Y.; data were analyzed by A. Ni, C.L.B., A.Y., F.S., V.E.S., and A.A.

### Statistical methods

Patient outcome was analyzed by OS, PFS, and EFS. OS was calculated either from time of diagnosis or from time of treatment commencement, as indicated on the figure, until last follow-up or death. PFS and EFS times for sequential lines of therapy were calculated from treatment commencement until qualifying event (progression or death for PFS; progression, change of treatment, or death for EFS). EFS12 failures were defined as patients with disease progression, change of therapy or death within 12 months of treatment initiation (EFS12). Date of progression was assessed based on chart review; for cases of unclear documentation, radiographic imaging was reviewed. Patients with inadequate response to treatment, change of treatment, or stable disease without subsequent documented progression were censored in the PFS analysis. Overall survival from time of transformation to diffuse large B-cell lymphoma (DLBCL) or DLBCL with features of Burkitt lymphoma was calculated from the time of first recorded pathologic transformation. OS, PFS, and EFS were evaluated using the Kaplan−Meier method. The OS, PFS, and EFS times for sequential lines of therapy were compared across lines of therapy using the log-rank test with adjustment for within-patient correlation. PFS and EFS were compared to other clinical variables using the log-rank test. Chi-squared method was used to compare PFS curves between groups by stage.

The risk of biopsy-proven transformation to DLBCL or DLBCL with features of Burkitt lymphoma was assessed using a competing risk analysis wherein patients can experience either transformation or death without transformation. Time origin was set at time of diagnosis; rates of transformation and death without transformation at specific time points were calculated.

We assessed patients’ FLIPI score at diagnosis and at initiation of first treatment^16,17^. In patients with incomplete FLIPI components, risk category was determined if omission of a component did not alter the risk category. Stable FLIPI was defined as retaining the original low or intermediate risk score between diagnosis and initiation of treatment (low to low, intermediate to intermediate). FLIPI was defined as increased between diagnosis and initiation of treatment if the risk category increased (low to intermediate, low to high, intermediate to high). This secondary FLIPI analysis excluded patients whose FLIPI score decreased or remained high risk from diagnosis to initiation of treatment (high to high, high to intermediate, high to low, intermediate to low). Overall survival was compared among the entire cohort as well as between the FLIPI categories (stable vs increased). Chi-squared test and Fisher’s exact test were used to compare categorical variables by FLIPI score change status.

### Data-sharing statement

For de-identified original data, please contact leec@mskcc.org. Requests would be submitted to our institutional review board for consideration and review prior to any data sharing.

## Results

### Patient characteristics

**Fig. 1:**
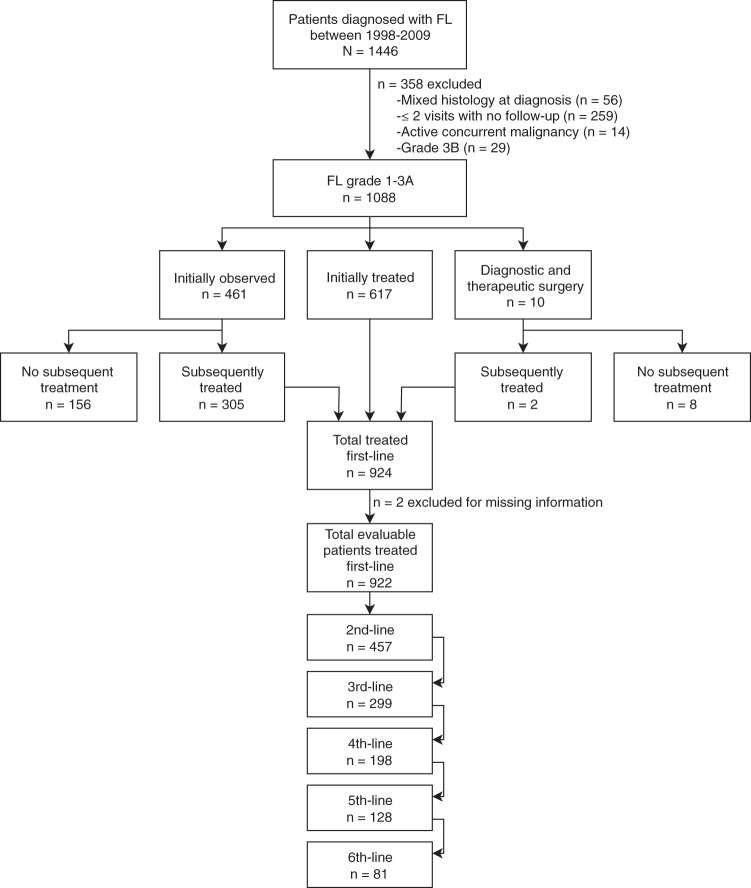
Flow diagram of FL patients included in our analysis. Patients diagnosed during the years 1998−2009 were assessed for eligibility. We analyzed 1088 patients while excluding 358 patients for active concurrent malignancy, a composite histology at diagnosis, grade 3B histology, or inadequate follow-up. Nine hundred and twenty-four patients were treated, either initially or after an observation period, and 164 patients never required treatment.

We identified 1446 consecutive adult patients (≥18 years) diagnosed with FL at MSK from January 1, 1998 to December 31, 2009. We excluded 358 patients who had active concurrent malignancy, grade 3B FL, mixed histology at initial diagnosis, or fewer than three visits to our institution (Fig. 1). Median age of the resulting 1088 patients was 57 years (range, 20–94) with median follow-up of 8.3 years (range, 0.2–17.5). Clinical characteristics are provided in Table 1. Two-thirds of patients had stage III or IV disease at diagnosis. Bone marrow biopsy results were available for 79% of patients; therefore, patients with stage III disease were provisionally staged dependent on status of bone marrow biopsy. FLIPI risk score at diagnosis was available for 851 (78%) patients, of whom almost one-third were low-risk. Positron emission tomography with 2-deoxy-2-[fluorine-18] fluoro-d-glucose integrated with computed tomography (PET/CT) was available for 60% of patients at diagnosis. Of the 924 patients who required treatment, 468 (51%) patients were managed with an anthracycline during their treatment course and 739 (80%) were treated with rituximab-containing therapy.

**Table 1:** Patient population.

Characteristic		Total (*n* = 1088)	Initially observed (*n* = 461)	Initially treated (*n* = 617)	Diagnostic and therapeutic surgery (*n* = 10)	Required treatment (*n* = 924)
Median age (range), years		57 (20–94)	57 (22–88)	57 (20–94)	67 (47–81)	57 (20–94)
Sex	Female	550 (51)	244 (53)	302 (49)	4 (40)	452 (49)
	Male	538 (49)	217 (47)	315 (51)	6 (60)	472 (51)
FLIPI at diagnosis	High	255 (23)	73 (16)	181 (29)	1 (10)	235 (25)
	Intermediate	259 (24)	126 (27)	131 (21)	2 (20)	229 (25)
	Low	337 (31)	148 (32)	184 (30)	5 (50)	266 (29)
	Unknown	237 (22)	114 (25)	121 (20)	2 (20)	194 (21)
Stage at diagnosis	I	199 (18)	51 (11)	140 (23)	8 (80)	171 (19)
	II	142 (13)	80 (17)	60 (10)	2 (20)	102 (11)
	III	317 (29)	168 (36)	149 (24)	0 (0)	270 (29)
	IV	420 (39)	154 (33)	266 (43)	0 (0)	372 (40)
	Unknown	10 (1)	8 (2)	2 (<1)	0 (0)	9 (1)
Hemoglobin, g/dl	≥12	829 (76)	373 (81)	450 (73)	6 (60)	697 (75)
	<12	114 (10)	26 (6)	85 (14)	3 (30)	101 (11)
	Unknown	145 (13)	62 (13)	82 (13)	1 (10)	126 (14)
Nodal area, cm^2^	≤4	666 (61)	289 (63)	367 (59)	10 (100)	541 (59)
	>4	404 (37)	160 (35)	244 (40)	0 (0)	365 (40)
	Unknown	18 (2)	12 (3)	6 (1)	0 (0)	18 (2)
LDH, U/L	Normal	620 (57)	276 (60)	338 (55)	6 (60)	525 (57)
	Elevated	162 (15)	40 (9)	121 (20)	1 (10)	149 (16)
	Unknown	306 (28)	145 (31)	158 (26)	3 (30)	250 (27)
Bone marrow	Negative	528 (49)	182 (39)	339 (55)	7 (70)	462 (50)
	Positive	327 (30)	129 (28)	198 (32)	0 (0)	293 (32)
	Unknown	233 (21)	150 (33)	80 (13)	3 (30)	169 (18)
PET staged	No	403 (37)	176 (38)	223 (36)	4 (40)	350 (38)
	Yes	652 (60)	269 (58)	378 (61)	5 (50)	542 (59)
	Unknown	33 (3)	16 (3)	16 (3)	1 (10)	32 (3)
Anthracycline exposure at any time	No	424 (39)	220 (48)	195 (32)	9 (90)	314 (34)
	Yes	468 (43)	140 (30)	327 (53)	1 (10)	467 (51)
	Unknown	196 (18)	101 (22)	95 (15)	0 (0)	143 (15)
Rituximab exposure at any time	No	193 (18)	20 (4)	164 (27)	9 (90)	185 (20)
	Yes	739 (68)	285 (62)	453 (73)	1 (10)	739 (80)
	Unknown	156 (14)	156 (34)	0 (0)	0 (0)	0 (0)

Data are *n* (%).

### Initial observation vs treatment

Of the 1088 patients included in this analysis, 461 were initially observed, 617 required initial therapy with systemic therapy or radiation, and 10 patients underwent a diagnostic and therapeutic surgery (Fig. 1). Eight of the 10 surgery patients never required additional treatment. In the initial observation group, 156 patients never required treatment. In total, 164/1088 (15%) patients never required treatment beyond their diagnostic and therapeutic procedure. We excluded two patients with incomplete first-line treatment information, leaving 922 patients who required systemic therapy or radiation and had available treatment data.

Among the patients who were initially observed rather than receiving immediate treatment (*n* = 461), median duration of observation was 3.9 years (95% CI, 3.4–4.5). For patients who were initially observed and subsequently treated (*n* = 305), median time to first treatment was 2.3 years (range, 0.27–13.33). In the initially treated population (*n* = 617), median time from diagnosis to first treatment was 0.14 year (95% CI, 0.13–0.15). Overall survival was not adversely affected by observation strategy (*p* = 0.206) (Supplementary Fig. 1).

### Survival outcomes by baseline characteristics

**Fig. 2:**
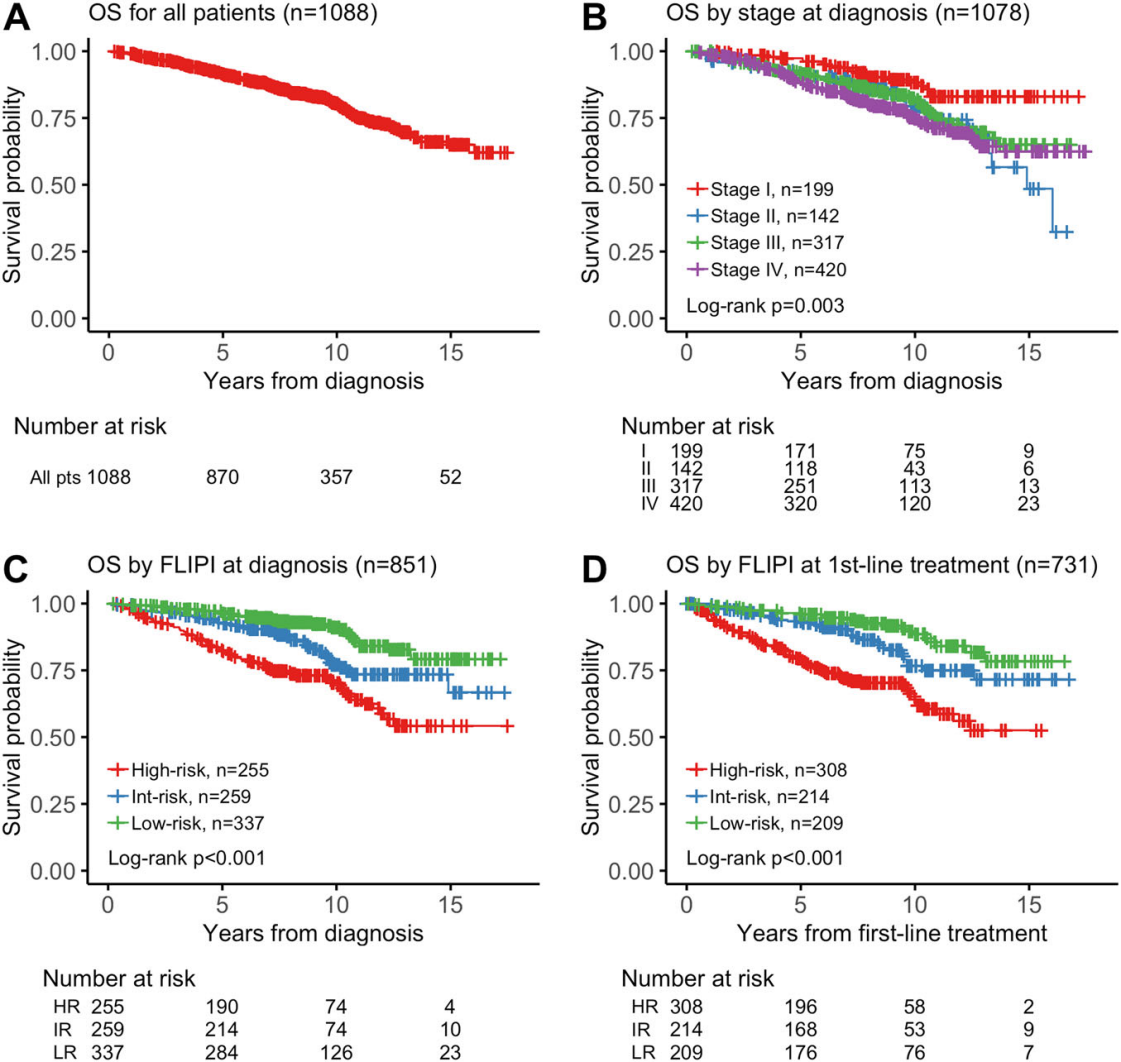
Survival outcomes. **a** OS from time of diagnosis for 1088 patients. **b** OS from time of diagnosis for 1078 patients based on stage at diagnosis. Ten patients excluded for an unknown stage at diagnosis. Stage III disease included patients with provisional stage III, as bone marrow biopsy was not performed for 21% of all patients. **c** OS from time of diagnosis for 851 patients with FLIPI score at time of diagnosis; FLIPI score at diagnosis unknown for 237 patients. **d** OS from time of first-line treatment for 731 patients with FLIPI score at time of first treatment; FLIPI score at treatment unknown for 191 patients while 2 patients had incomplete data for survival analysis.

Median OS was not reached (Fig. 2a), based on 208 observed deaths. Overall survival for all 1088 analyzed patients was 92% at 5 years (95% CI, 0.90–0.93), 80% at 10 years (95% CI, 0.78–0.83), and 65% at 15 years (95% CI, 0.60–0.71). We observed 5-year OS of 97% for stage I patients, 91% for stage II, 92% for stage III, and 88% for stage IV (Fig. 2b). FLIPI at diagnosis and at first treatment was prognostic for OS (Fig. 2c, d). Ten-year OS rates for patients with low, intermediate, and high FLIPI score at diagnosis were 91%, 77%, and 70%, respectively.

### Survival outcomes by time period

Management of FL has evolved over the years; therefore, we evaluated the effect of modern FL treatments on clinical outcomes. Patients were distributed between three time frames across two decades: 1998–2000 (*n* = 203), 2001–2005 (*n* = 448), and 2006–2009 (*n* = 437). We selected the initial period of 1998–2000 assuming that time was needed for rituximab adoption in the community; the two subsequent time periods divided the following decade. Median OS was not reached at any of the three time frames (log-rank *p* = 0.144) (Supplementary Fig. 2A). Overall survival did not significantly increase over time: OS 5 years after diagnosis was 90.8% (95% CI, 86.8–94.9%), 90.9% (95% CI, 88.1–93.7%), and 92.7% (95% CI, 90.2–95.2%), respectively, for patients diagnosed between 1998–2000, 2001–2005, and 2006–2009. Similarly, 10-year OS was 76.3% (95% CI, 70.2–82.9%), 81.0% (95% CI, 77.1–85.1%), and 84.0% (95% CI, 79.7–88.6%), respectively, for patients diagnosed between 1998–2000, 2001–2005, and 2006–2009. Progression-free survival was shorter in the first time frame than in the latter two, with median PFS of 2.5 years (95% CI, 2.1–4.4 years), 6.0 years (95% CI, 4.4–8.7 years), and 4.8 years (95% CI, 3.7–6.7), respectively, for diagnosis from 1998–2000, 2001–2005, and 2006–2009 (*p* = 0.003) (Supplementary Fig. 2B). Five-year PFS from time of first-line treatment was 40.5% (95% CI, 33.7–48.5%), 53.6% (95% CI, 48.6–59.2%), and 48.4% (95% CI, 43.1–54.4%), respectively, for patients diagnosed between 1998–2000, 2001–2005, and 2006–2009.

### Patterns of treatment in FL

Among the 922 patients who received treatment, first-line therapy was rituximab in combination with chemotherapy in 52.1% (*n* = 480) and chemotherapy alone in 10.3% (*n* = 95) (Supplementary Table 1). Anthracycline-based chemotherapy (with or without rituximab) represented 45% of the first-line treatment. Rituximab single-agent therapy and radiation alone were first-line treatment in 18.1% (*n* = 167) and 15.5% (*n* = 143) of the population, respectively. Recurrent uses of single-agent rituximab and radiotherapy were common throughout multiple lines of therapy. Alkylator-based chemotherapy represented 22.3% of second-line therapy. Radioimmunotherapy made up 3–9% of treatments in second- to sixth-line therapy. Ten percent of treated patients (91/922) received stem cell transplants during their course of therapy, 6% (*n* = 54) autologous stem cell transplant and 4% (*n* = 37) allogeneic stem cell transplant. Investigational therapies were uncommon in first-line therapy but increased to 8–22% with increasing lines of therapy.

### Transformation

For our cohort of 1088 patients, transformation was observed in 167 (15.3%) patients (median follow-up 8.3 years (range: 0.2–17.5)). Follicular lymphoma most commonly transformed to diffuse large B-cell lymphoma (DLBCL) or DLBCL with features of Burkitt lymphoma (*n* = 164); however, one patient had a later diagnosis of peripheral T-cell lymphoma and two patients had later diagnoses of marginal zone lymphoma. Of the 164 patients who transformed to DLBCL or DLBCL with features of Burkitt lymphoma, transformation occurred prior to therapy in 19% (31/164) and after first-line therapy in 81% (133/164) of patients. For patients with a transformation event after first-line therapy but after diagnosis of FL, anthracycline was a component of prior therapy in 71/133 (53%) events. Competing risk analysis showed risk of histological transformation at 2, 5, and 10 years after diagnosis to be 3%, 8%, and 16%, respectively. Risk of death without transformation at 2, 5, and 10 years after diagnosis was 2%, 6%, and 14%, respectively (Supplementary Fig. 3A).

We compared OS in patients whose transformation event was before or after first-line therapy. The latter was associated with increased risk of death (HR 3.35; 95% CI, 1.34–8.39; *p* = 0.010) (Supplementary Fig. 3B). Median OS after transformation was not reached (95% CI, 10.1 years − not reached [NR]) in patients who transformed prior to frontline therapy (*n* = 31), whereas median OS in patients who transformed after frontline therapy (*n* = 133) was 7.6 years (95% CI, 2.4-NR; log-rank *p* = 0.01). Five-year OS from time of transformation was 83% (95% CI, 61–93%) for patients who transformed prior to frontline therapy and 55% (95% CI, 45–64%) for patients who transformed after frontline therapy.

### OS, PFS, and EFS by line of therapy

**Fig. 3:**
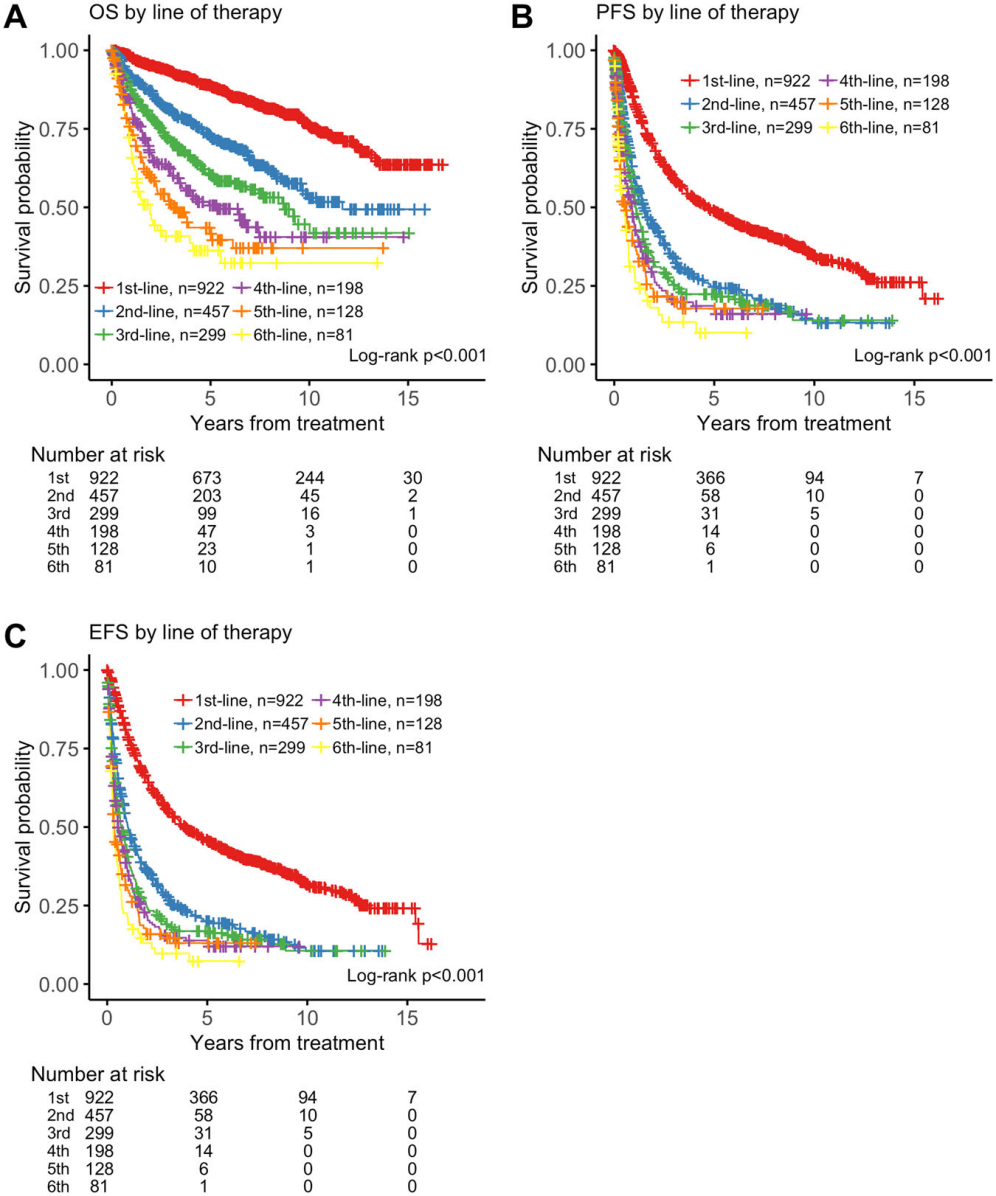
OS, PFS, and EFS outcomes based on lines of therapy for 922 patients. **a** OS from commencement of line of therapy. **b** PFS from commencement of line of therapy. **c** EFS from commencement of line of therapy.

We evaluated 922 patients for OS, PFS, and EFS by line of therapy. Despite FL treatment options increasing between 1998 and 2009, individual patients’ treatment outcomes nevertheless declined with increasing lines of therapy (Fig. 3a). Among the 922 patients receiving first-line therapy, median OS was not reached (Table 2). After second-line therapy, median OS was 11.67 years (95% CI, 9.67−NR). Median OS further decreased with each line of therapy and decreased to 3.13 years (95% CI, 2.22–6.13) after fifth-line therapy. At four or more lines of therapy, median PFS was 0.9 years (95% CI, 0.6–1.1), and median EFS was 0.6 years (95% CI, 0.5–0.8) (Fig. 3b, c).

**Table 2:** Outcomes of OS, PFS, and EFS by lines of therapy.

	1st-line (*n* = 922)	2nd-line (*n* = 457)	3rd-line (*n* = 299)	4th-line (*n* = 198)	5th-line (*n* = 128)	6th-line (*n* = 81)
OS	NR (NR-NR)	11.67 (9.67−NR)	8.75 (6.84−NR)	5.34 (3.51−NR)	3.13 (2.22–6.13)	1.93 (1.25–5.52)
PFS	4.73 (3.93–5.71)	1.51 (1.22–1.92)	1.07 (0.93–1.39)	0.90 (0.59–1.10)	0.55 (0.33–0.92)	0.48 (0.28–0.71)
EFS	3.91 (3.39–4.79)	1.04 (0.89–1.31)	0.73 (0.57–0.94)	0.56 (0.48–0.84)	0.35 (0.29–0.62)	0.30 (0.26–0.50)

Data are median (95% CI) years.

*NR* not reached.

PFS and EFS outcomes after first-line therapy were affected by stage at treatment: patients with stage I FL enjoyed a prolonged remission after first-line therapy (Supplementary Table 2). Median PFS was 8.8 years (95% CI, 6.7–11.4) for stage I patients vs <5.1 years for stage II−IV patients (chi-squared *p* = 0.006). EFS after first-line therapy was also statistically significantly longer for stage I patients (chi-squared *p* = 9 × 10^–4^). However, stage at first treatment was not prognostic for PFS or EFS following second-line or later therapy (Supplementary Table 2).

### Prognostic value of changes in FLIPI score at diagnosis and treatment

**Fig. 4:**
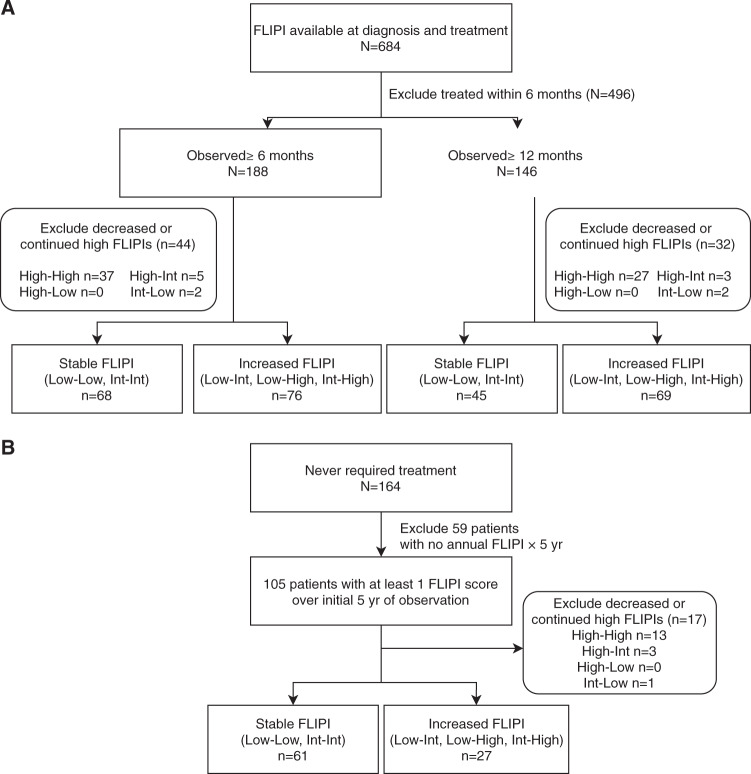
Flow diagram of patients for stable vs increased FLIPI analysis. **a** Treated patients with FLIPI score at time of diagnosis and treatment (*n* = 684) were excluded if they were treated within 6 months or had unknown treatment dates (*n* = 496). Stable FLIPI included patients whose FLIPI risk category did not change between diagnosis and first treatment (low to low, or intermediate to intermediate). Increased FLIPI included patients whose FLIPI score increased between diagnosis and first treatment (low to intermediate, low to high, or intermediate to high). We excluded patients whose FLIPI score decreased between diagnosis and first treatment or remained high (high to high, high to intermediate, high to low, or intermediate to low). Patients observed ≥6 months (*n* = 188) and ≥12 months (*n* = 146) were evaluated for stable or increased FLIPI scores. **b** Patients who never required treatment were evaluated using FLIPI at diagnosis and the highest FLIPI during the initial 5 years of observation to obtain their status of stable or increased FLIPI.

Advanced-stage FL is commonly managed with active observation. We sought to understand the clinical impact when FLIPI worsens during observation, between diagnosis and initial treatment. We identified 684 patients with FLIPI available at diagnosis and first treatment and excluded 496 patients who initiated therapy within 6 months (Fig. 4a). Of the remaining 188 patients who were observed >6 months, 68 (36%) patients had stable FLIPI, 76 (40%) patients had increased FLIPI, and 44 (23%) patients were excluded for decreased or continued high FLIPI. Of the 146 patients observed ≥12 months, 45 (31%) patients had stable FLIPI, 69 (47%) patients had increased FLIPI, and 32 (22%) patients were excluded for decreased or continued high FLIPI.

For the 164 patients who never required therapy, we reviewed medical records to determine FLIPI scores for the first 5 years of their observation (Fig. 4b). We were able to ascertain FLIPI score from diagnosis and at least one other time point for 105 patients. For patients with multiple FLIPI scores over the 5-year period, the maximum FLIPI was used for comparison to FLIPI at diagnosis. Sixty-one (58%) of these patients had a stable FLIPI, 27 (26%) patients had a increased FLIPI, and 17 (16%) patients were excluded for decreased or continued high FLIPI. Among the 164 patients, median duration of follow-up was 7.3 years (range, 0.2–16.7 years).

Among the 144 patients who were observed ≥6 months who eventually required therapy and had stable or increased FLIPI (Fig. 4a), median time to first treatment was 1.6 years (range, 0.5–12.5) for patients with stable FLIPI (*n* = 68) and 3.7 years (range, 0.5–13.3) for patients with increased FLIPI (*n* = 76), Wilcoxon test *p* < 0.001. For the 114 patients who were observed ≥12 months, eventually required therapy, and had stable or increased FLIPI, median time to first treatment was 2.9 years (range, 1.0–12.5) for patients with stable FLIPI (*n* = 45) and 3.9 years (range, 1.1–13.3) for patients with increased FLIPI (*n* = 69), Wilcoxon test *p* = 0.002.

We sought to understand the causes of FLIPI score changes by analyzing the components contributing to the change. For patients initially observed before treatment, increased FLIPI was driven by increased nodal involvement in 41/76 (54%) observed ≥6 months and 36/69 (52%) observed ≥12 months. Other contributing factors were increased stage (in 31/76 [41%] patients observed ≥6 months and 30/45 [67%] observed ≥12 months); abnormal LDH (26/71 [37%] observed ≥6 months and 24/64 [38%] observed ≥12 months); increasing age (21/76 [28%] observed ≥6 months and 21/69 [30%] observed ≥12 months); and reduced hemoglobin (19/70 [27%] observed ≥6 months and 19/64 [30%] observed ≥12 months).

**Fig. 5:**
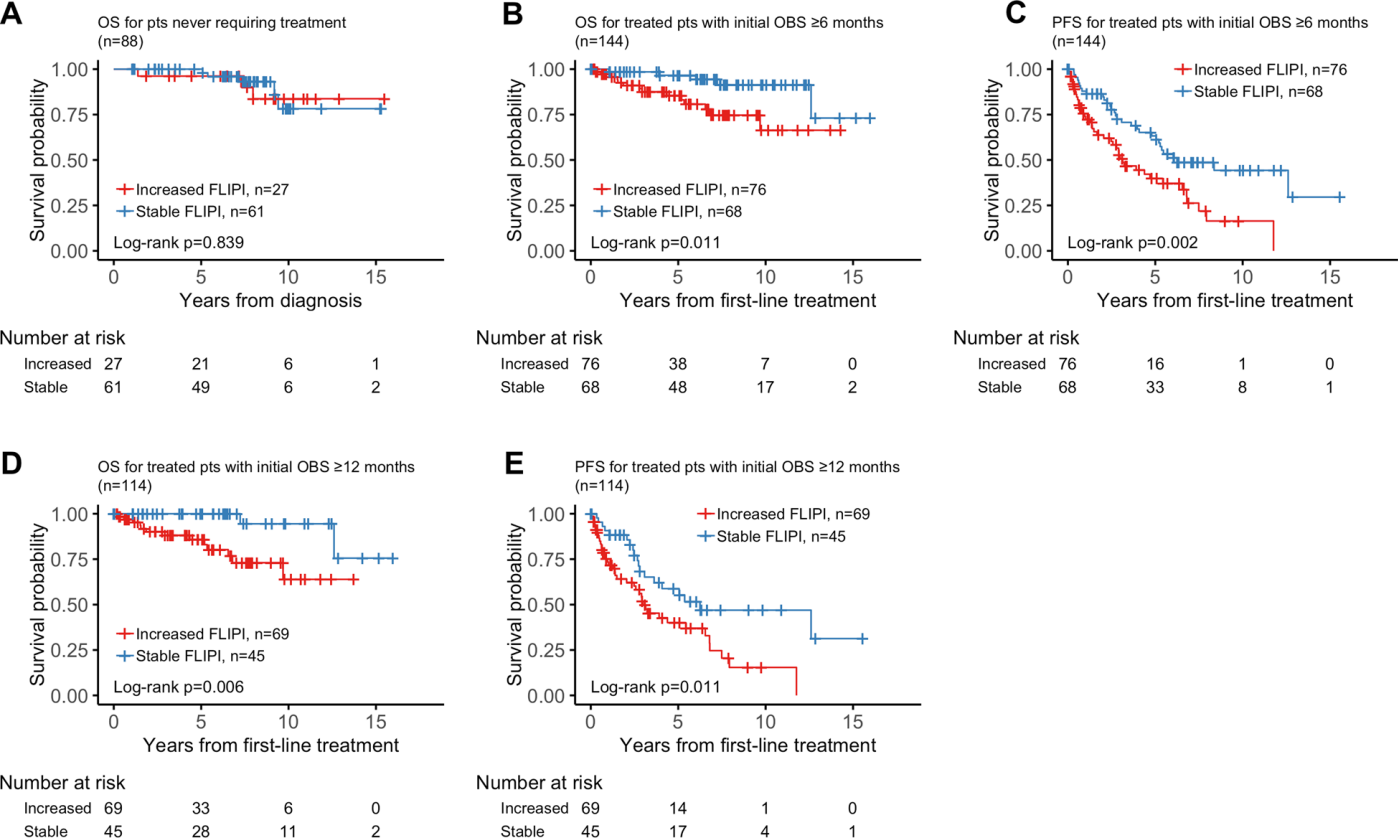
Comparison of outcomes for patients with stable vs dynamic FLIPI. **a** OS from time of diagnosis in patients who were never treated and had an annual FLIPI score within first 5 years of observation. **b** OS since time of first-line treatment in treated patients observed ≥6 months. **c** PFS since time of first-line treatment in treated patients observed ≥6 months. **d** OS since time of first-line treatment in treated patients observed ≥12 months. **e** PFS since time of first-line treatment in treated patients observed ≥12 months.

Among the 88 patients who never required therapy and had available FLIPI within a 5-year period, no differences in OS were observed between patients with stable (*n* = 61) vs increased FLIPI (*n* = 27) (Fig. 5a). Factors contributing to increased FLIPI were increased stage in 12/27 cases (44%), increased nodal areas in 9/27 cases (33%), increased age in 9/27 cases (33%), abnormal LDH in 6/27 cases (22%), and reduced hemoglobin in 3/27 cases (11%).

In patients observed ≥6 months from their diagnosis, increased FLIPI was associated with an inferior OS (*p* = 0.011) (Fig. 5b). Median OS was not reached for either the increased or stable FLIPI groups. PFS was also notable for a negative association with increased FLIPI (*p* = 0.002) (Fig. 5c). The median PFS was 3.14 years (95% CI, 2.48–6.79) in the increased FLIPI group and 6.25 years (95% CI, 5.05−NR) in the stable FLIPI group. Similarly, in patients who were observed ≥12 months, increased FLIPI was a marker for inferior OS and PFS. While the median OS was not reached for either increased or stable FLIPI groups, OS was statistically significantly different (log rank *p* = 0.006) (Fig. 5d). The median PFS was 2.98 years (95% CI, 2.48–6.79) in the increased FLIPI group and 6.25 years (95% CI, 3.62−NR) in the stable FLIPI group (log-rank *p* = 0.011) (Fig. 5e).

Increased FLIPI during observation was also associated with a higher rate of EFS12 failure after treatment initiation. For 76 patients who were observed ≥6 months and subsequently treated with increased FLIPI, 21 (27.6%) failed EFS12 after treatment. In comparison, for 68 patients who were treated with stable FLIPI after ≥6 months of observation, only 8 (11.8%) failed EFS12. Similarly, the EFS12 failure rates in patients with increased or stable FLIPI after ≥12 months of observation were 27.5% (19/69) and 8.9% (4/45), respectively (Supplementary Table 3).

In patients observed without therapy, clinical parameters seen at diagnosis such as SUV, Ki-67, and abnormal LDH were similar across all groups (Supplementary Table 4). Patients with increased vs stable FLIPI were similarly treated with rituximab at first-line therapy. Patients with increased FLIPI had a higher risk of transformation compared to patients with stable FLIPI. Using a competing risk analysis, in patients who were observed ≥6 months, transformation rate at 10 years after diagnosis was 28.1% in patients with increased FLIPI vs 14.5% in patients with stable FLIPI. Similarly, in patients who were observed ≥12 months, transformation rate at 10 years after diagnosis was 27.6% in patients with increased FLIPI vs 14.4% in patients with stable FLIPI.

## Discussion

Despite the commonly indolent nature of FL, a subpopulation of patients harbors aggressive disease. Moreover, the heterogenous nature of FL makes approval of novel therapies challenging. Therefore, the identification of patients with high-risk biology is important for the development of future treatments. Existing markers of high risk include failure to achieve a complete response to initial chemoimmunotherapy, and retreatment within 2 years of initial treatment. In this study, we describe the outcomes of patients with FL treated at our center, with the aim of providing additional biomarkers to identify high-risk patients for early treatment intervention. This population was diagnosed after FDA approval of rituximab; therefore, rituximab was readily available to manage the disease. Despite this, 20% (185/922) of the patients who required therapy never had rituximab exposure throughout their course of therapy; however, 52% (97/185) of these patients were treated with radiotherapy.

We found outcomes of this generation of patients are improving. Median OS for all patients has not been reached even with nearly 20 years follow-up on the earlier patients. Five-year OS was 92% for all patients, highlighting the good outcomes of most patients with FL. Progression-free survival improved during the study period, likely through improved therapies. This study supports earlier studies demonstrating patients receiving initial observation have the same OS as those treated promptly after diagnosis^15,18,19^.

The Stanford University FL experience demonstrated improved OS over multiple eras, with stable PFS of approximately 2 years across all eras between 1960 and 2003^20^. Our study reports median OS is not reached for patients diagnosed during time periods 1998–2000, 2001–2005, and 2006–2009; and median PFS improved from 2.5 years for 1998–2000 to a range of 4.8–6.0 years in the latter two time frames. We note this improved PFS despite the lack of maintenance rituximab in 80% of our patients^6^. We found 5-year PFS for patients after first-line therapy to range from 40 to 55%. In comparison, the PRIMA study demonstrated a 6-year PFS estimate of 43% in the no-maintenance arm and 69% in the rituximab maintenance arm^11,12^. The FOLL05 study of advanced-stage FL did not incorporate maintenance rituximab and had an 8-year PFS of 48%^7^. The StiL study demonstrated median PFS not reached, with median 3.75 years of follow-up in R-bendamustine arm and 3.4 years in the RCHOP arm^10^.

Transformation to aggressive lymphoma has been linked to adverse prognosis^21–26^. Similar to results from the PRIMA trial where 194/1018 (19%) patients had documented histologic transformation with a median follow-up of 6 years^21^, we identified 167 histologic transformation events in 1088 patients (15%) with a median follow-up of 8.9 years. The data support an adverse prognosis in patients with FL who transform after first-line therapy. In PRIMA, 5-year OS of the transformed population was approximately 40% at 5 years^21^, similar to our 5-year OS estimate of 55% in patients who transformed after first-line therapy.

Outcomes in FL after multiple lines of therapy have been incompletely described in the literature, another reason for our current study. Follicular lymphoma is typically responsive to frontline chemotherapy but may later become refractory^27,28^. Prior to the widespread use of rituximab, retreatment after multiple lines of therapy was required every 2.75 years and median OS after first relapse was 5 years^27^. The development of more effective FL therapies has improved outcomes. In our dataset, median OS for patients who received second-line treatment was >10 years.

The availability of new treatments for FL also reflects a need for new benchmarks to identify effective treatment strategies. In many tumors, improvement in OS remains the gold standard for clinical approval; however, the long heterogenous course of FL makes OS a challenging primary endpoint. Progression at 24 months is a potential endpoint for FL; however, its impact on prognosis is less validated^29–31^. PFS remains a reasonable clinical trial endpoint. We demonstrate that at fourth-line therapy and beyond, PFS is less than 1 year, providing a clinical endpoint that is robust and feasible to assess within a clinical trial context. This is supported by the National LymphoCare study that also showed PFS decreasing with increasing lines of therapy: 6.6, 1.5, 0.83, 0.69, and 0.68 years after first, second, third, fourth, and fifth-line therapy, respectively^13^. Both our and the LymphoCare analyses provide a benchmark for future drug approval in relapsed FL.

This study also assessed the impact of changes to the FLIPI score during observation as a marker for adverse prognosis. Patients observed for ≥6 or ≥12 months were evaluated for increased FLIPI between diagnosis and prior to first-line treatment. We found that subsequently treated patients whose FLIPI increased during observation had inferior OS and PFS. FLIPI increase was most commonly associated with increased nodal involvement, progression to advanced stage, and abnormal LDH. Of course, the increased FLIPI population may reflect patients who had adverse biology at diagnosis, especially since we continue to demonstrate that observation had no impact on OS. Nonetheless, in this era of readily available, easily tolerated therapies, it may be questionable whether initial observation is the ideal strategy. Future randomized trials comparing observation to upfront treatment may be warranted.

The strength of this analysis lies in the large cohort. However, it is limited by its single-center and retrospective nature. Though our patients received heterogenous treatments reflecting a real-world strategy, they lack the greater diversity of patients treated by multiple unaffiliated practices.

This study benchmarks a single institution’s outcomes of FL in the post-rituximab era. The work identifies higher number of lines of therapy and increased FLIPI score as markers for high-risk biology in FL. Based on EFS and PFS observed in this large retrospective series, we can consider designing clinical trials for fourth-line treatment of FL. Future prospective studies are needed to assess a possible correlation between increased FLIPI score and inferior outcomes.

## Supplementary information

Supplementary information
