# Supplementary material for: Follicular lymphoma in the modern era: survival, treatment outcomes, and identification of high-risk subgroups
Source: Blood Cancer J. 2020 Jul 17;10(7):74. doi: 10.1038/s41408-020-00340-z (PMC7366724; doi:10.1038/s41408-020-00340-z)

**SUPPLEMENTAL**

**Supplemental Table 1.** Patterns of treatment in FL. Types of treatment from first-line to sixth-line therapy characterized.

**Supplemental Table 2.** PFS and EFS outcomes by lines of therapy, based on stage at first-line treatment.

**Supplemental Table 3.** EFS12 failure rate in patients who were observed ≥6 months or ≥12 months, based on FLIPI status.

**Supplemental Table 4.** Characteristics of patients observed ≥6 months, ≥12 months or never treated, based on FLIPI status.

**Supplemental Figure 1.** **Overall survival of patients initially observed and requiring treatment.**

**Supplemental Figure 2.** **OS from time of diagnosis and PFS from time of first-line treatment, stratified by era of diagnosis.** (A) OS for all patients diagnosed between 1998-2000, 2001-2005, and 2006-2009. (B) PFS for all patients diagnosed between 1998-2000, 2001-2005, and 2006-2009.

**Supplemental Figure 3.** **Transformation risk assessment.** Of 1088 patients, transformation to diffuse large B cell lymphoma (DLBCL) occurred in 164 patients. (A) Competing risk assessment showed risk of death without transformation and risk of transformation.
(B) Transformation event relative to treatment timing was compared in an overall survival curve. Transformation occurring after first-line therapy was associated with an increased risk of death (HR 3.35; 95% CI, 1.34-8.39; p=0.010).

**Supplemental Table 1.** Patterns of treatment in FL. Types of treatment from first-line to sixth-line therapy characterized.


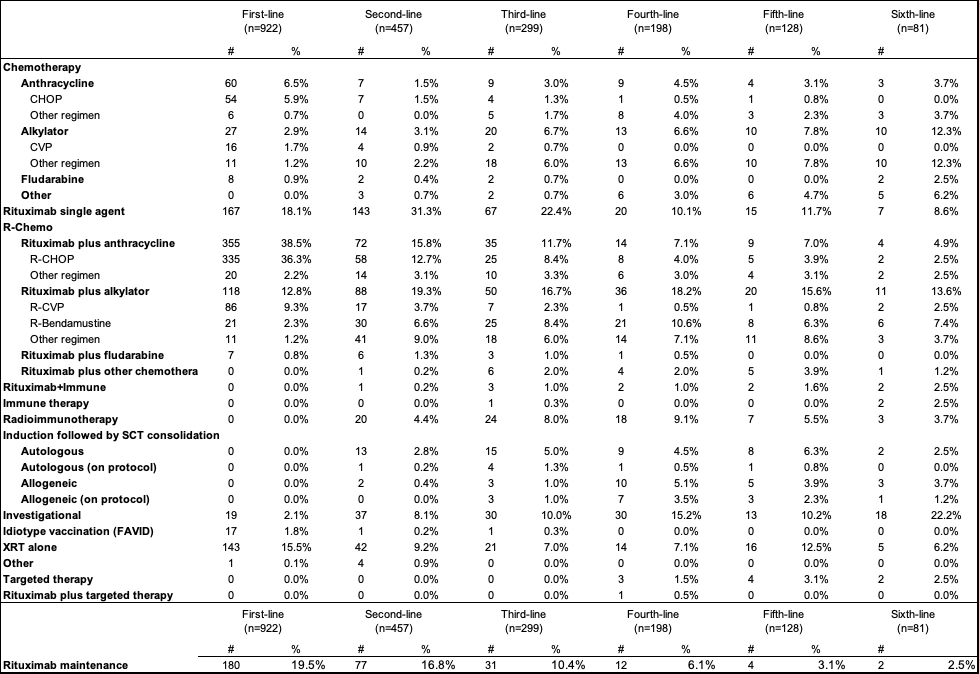


**Supplemental Table 2.** PFS and EFS outcomes by lines of therapy, based on stage at first-line treatment.

|  | 1st-line | 2nd-line | 3rd-line | 4th-line | 5th-line | 6th-line |
| --- | --- | --- | --- | --- | --- | --- |
| Stage I at first-line treatment | | | | | | |
| n | 138 | 45 | 23 | 9 | 4 | 1 |
| PFS | 8.76 (6.66-11.44) | 1.25 (0.91-3.08) | 1.34 (0.96-NR) | 0.32 (0.04-NR) | 0.31 (0.00-NR) | NR (NR-NR) |
| EFS | 8.76 (6.37-11.44) | 1.25 (0.91-3.08) | 1.11 (0.93-NR) | 0.32 (0.04-NR) | 0.31 (0.00-NR) | NR (NR-NR) |
| Stage II at first-line treatment | | | | | | |
| n | 75 | 33 | 12 | 9 | 7 | 5 |
| PFS | 5.05 (2.28-9.99) | 1.94 (0.81-NR) | 1.26 (0.41-NR) | 1.69 (0.50-NR) | 1.60 (0.08-NR) | 0.31 (0.08-NR) |
| EFS | 4.41 (2.21-9.99) | 1.94 (0.81-NR) | 0.73 (0.38-NR) | 0.90 (0.23-NR) | 0.31 (0.08-NR) | 0.20 (0.08-NR) |
| Stage III at first-line treatment | | | | | | |
| n | 268 | 138 | 98 | 66 | 39 | 23 |
| PFS | 4.08 (3.45-6.16) | 1.58 (1.41-2.23) | 0.82 (0.62-1.44) | 1.01 (0.63-2.02) | 0.41 (0.24-1.40) | 0.42 (0.14-1.56) |
| EFS | 3.53 (2.89-4.75) | 1.41 (1.00-1.58) | 0.62 (0.43-0.82) | 0.73 (0.38-1.24) | 0.41 (0.24-1.22) | 0.21 (0.14-0.70) |
| Stage IV at first-line treatment | | | | | | |
| n | 437 | 239 | 164 | 112 | 77 | 51 |
| PFS | 3.64 (2.98-5.00) | 1.27 (0.93-2.22) | 1.19 (0.94-1.49) | 0.76 (0.56-1.08) | 0.64 (0.33-1.23) | 0.55 (0.29-0.99) |
| EFS | 3.08 (2.58-3.95) | 0.80 (0.62-1.05) | 0.73 (0.57-1.08) | 0.56 (0.41-0.79) | 0.35 (0.29-0.68) | 0.33 (0.28-0.62) |

PFS and EFS data are median (95% CI) years.

NR, not reached.

**Supplemental Table 3.** EFS12 failure rate in patients who were observed ≥6 months or ≥12 months, based on FLIPI status.

|  | | EFS12 achiever | EFS12 failure | EFS12 Not Evaluable | P-value |
| --- | --- | --- | --- | --- | --- |
| Observed ≥ 6months | Increased FLIPI (N=76) | 46 | 21 | 9 | 0.011 |
|  | Stable FLIPI (N=68) | 58 | 8 | 2 |  |
| Observed ≥12 months | Increased FLIPI (N=69) | 42 | 19 | 8 | 0.009 |
|  | Stable FLIPI (N=45) | 39 | 4 | 2 |  |

**Supplemental Table 4.** Characteristics of patients observed ≥6 months, ≥12 months or never treated, based on FLIPI status.

|  | Observed ≥6 months | | Observed ≥12 months | | Never treated | |
| --- | --- | --- | --- | --- | --- | --- |
|  | Stable FLIPI  (n=68) | Increased FLIPI  (n=76) | Stable FLIPI  (n=45) | Increased FLIPI  (n=69) | Stable FLIPI  (n=61) | Increased FLIPI  (n=27) |
| Sex |  |  |  |  |  |  |
| Female | 24 (35) | 45 (59) | 17 (38) | 41 (59) | 29 (48) | 14 (52) |
| Male | 44 (65) | 31 (41) | 28 (62) | 28 (41) | 32 (52) | 13 (48) |
| Median age (range), years | 51 (22-80) | 57 (25-81) | 52 (22-78) | 57 (25-81) | 54 (35-81) | 58 (35-78) |
| Rituximab monotherapy at first-line therapy | 27 (40) | 33 (43) | 22 (49) | 30 (43) | NA | NA |
| Transformation |  |  |  |  |  |  |
| 5-year transformation rate after diagnosis* (%) | 4.4 | 12.3 | 6.7 | 10.5 | NA^†^ | NA^†^ |
| 10-year transformation rate after diagnosis* (%) | 14.5 | 28.1 | 14.4 | 27.6 | NA^†^ | NA^†^ |
| Diagnostic parameters |  |  |  |  |  |  |
| Median SUV (range) | 6.2 (2.0-19.4) | 5.4 (1.3-16.4) | 6.3 (2.0-19.4) | 5.4 (1.3-14.2) | 5.7 (0.9-19.4) | 6.4 (4.1-12.2) |
| Median Ki67 (range) | 0.2 (0.05-0.70) | 0.1 (0.05-0.60) | 0.15 (0.05-0.45) | 0.08 (0.05-0.60) | 0.13 (0.05-0.75) | 0.09 (0.02-0.75) |
| Abnormal LDH, n/N (%), U/L | 2/65 (3) | 7/74 (9) | 2/42 (5) | 6/67 (9) | 4/55 (7) | 1/27 (4) |
| Data are n (%), unless otherwise noted.  NA, not applicable; SUV, standardized uptake values.  *Based on competing risk analysis.  ^†^Never-treated patients would not undergo transformation and were not included in competing risk analysis. | | | | | | |

**Supplemental Figure 1.** **Overall survival of patients initially observed and requiring treatment.**


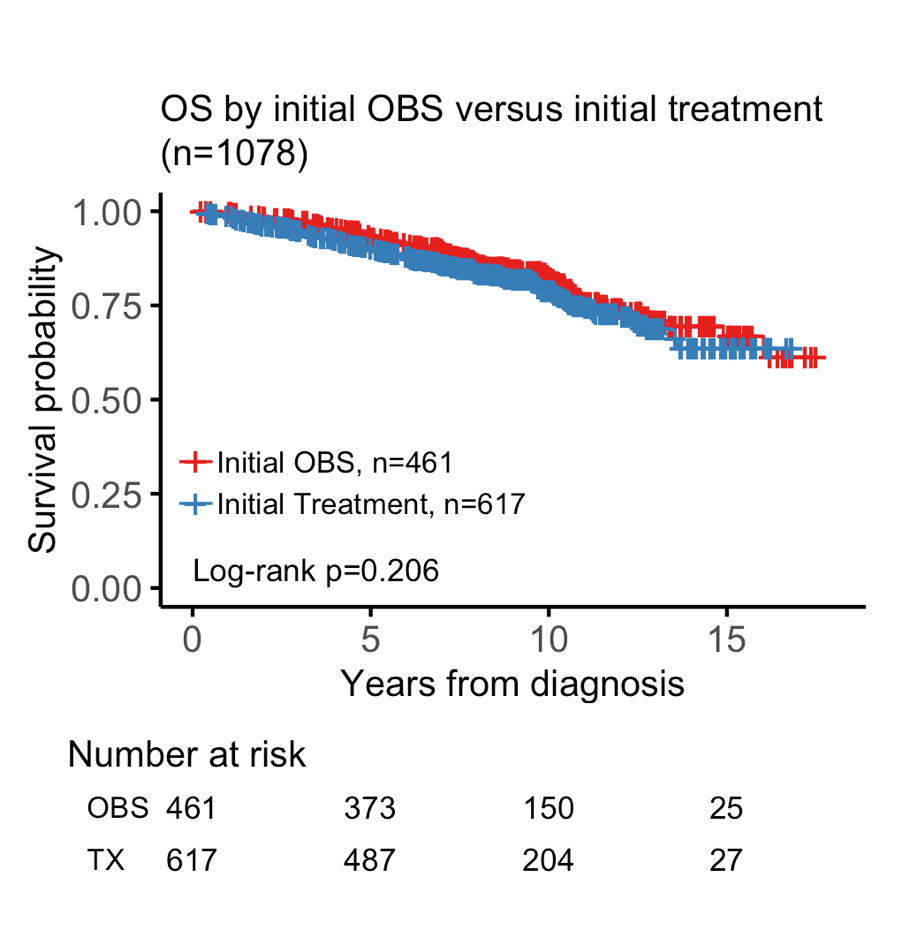


**Supplemental Figure 2.** **OS from time of diagnosis and PFS from time of first-line treatment, stratified by era of diagnosis.** (A) OS for all patients diagnosed between 1998-2000, 2001-2005, and 2006-2009. (B) PFS for all patients diagnosed between 1998-2000, 2001-2005, and 2006-2009.


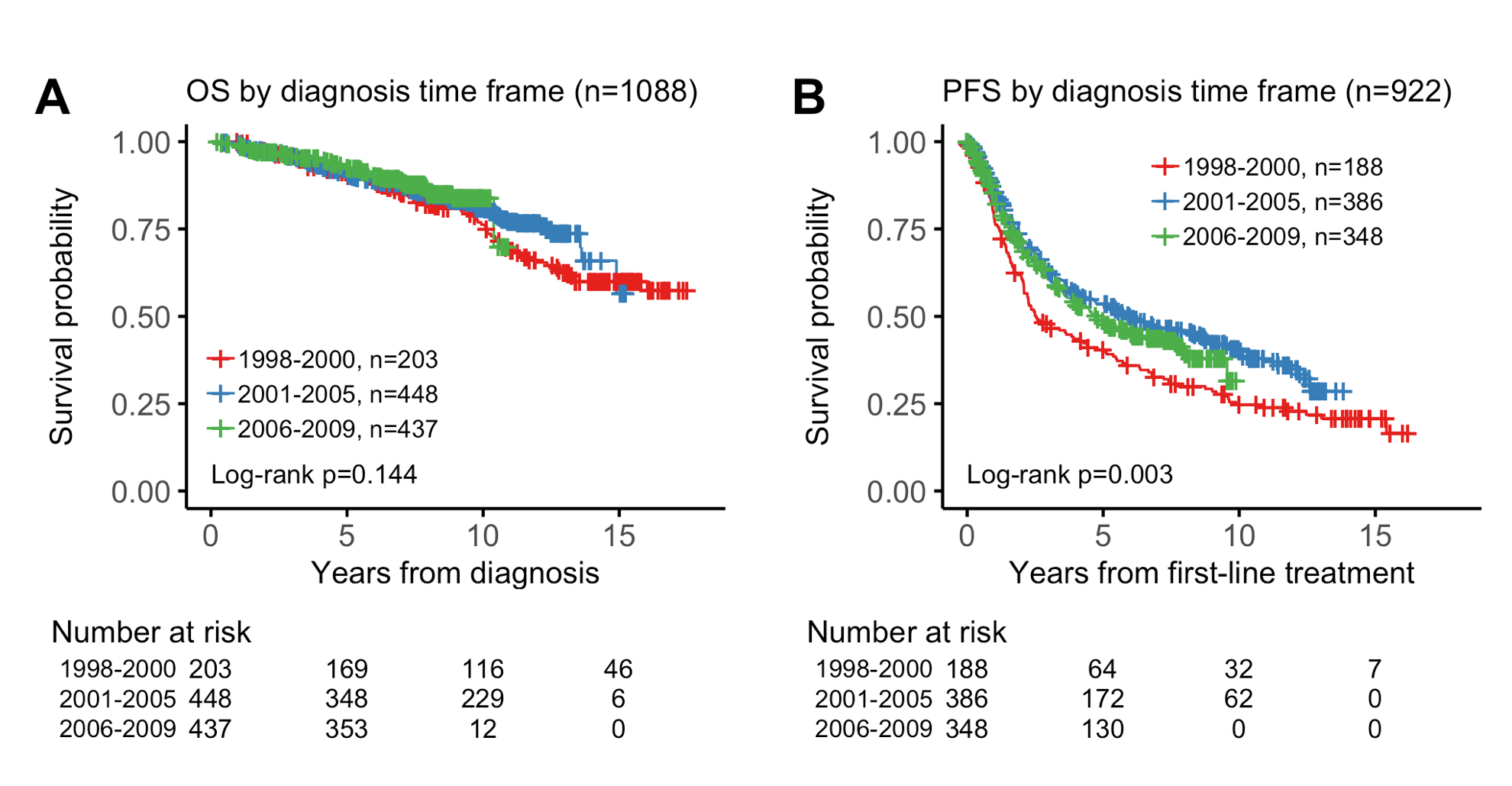


**Supplemental Figure 3.** **Transformation risk assessment.** Of 1088 patients, transformation to diffuse large B cell lymphoma (DLBCL) occurred in 164 patients. (A) Competing risk assessment showed risk of death without transformation and risk of transformation.
(B) Transformation event relative to treatment timing was compared in an overall survival curve. Transformation occurring after first-line therapy was associated with an increased risk of death (HR 3.35; 95% CI, 1.34-8.39; p=0.010).


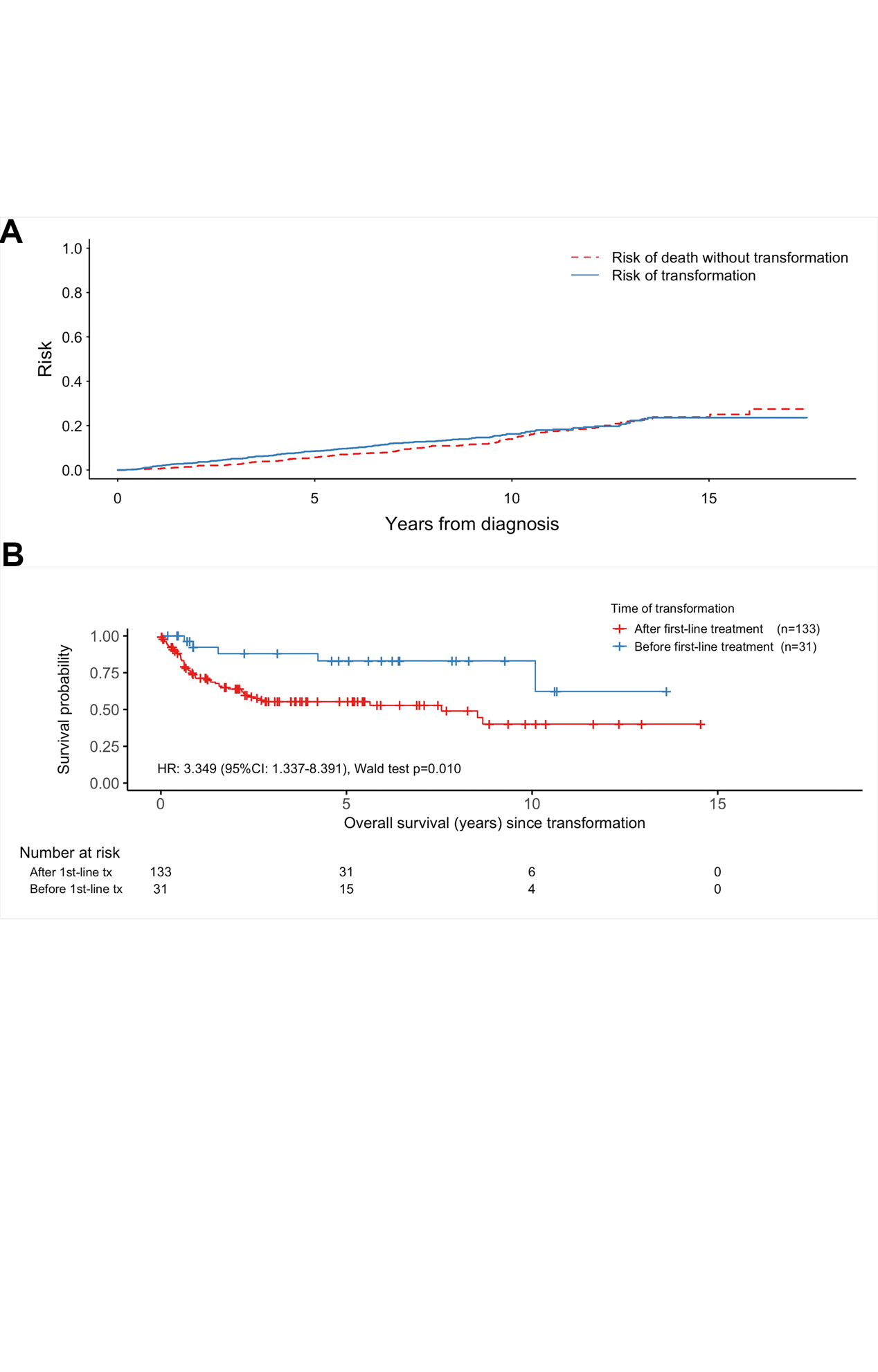

Supplement: Supplementary file 1 — Supplementary information [file 41408_2020_340_MOESM1_ESM.docx]
